# EXAMINING A RANDOMLY ASSIGNED MUSIC PROGRAM ON STRESS AND WELLNESS: CARE PARTNERS OF OLDER ADULTS LIVING WITH DEMENTIA

**DOI:** 10.1093/geroni/igad104.0337

**Published:** 2023-12-21

**Authors:** Catherine Tompkins, Emily Ihara, Shannon Layman, Megumi Inoue, Gilbert Gimm

**Affiliations:** George Mason University, Fairfax, Virginia, United States; George Mason University, Fairfax, Virginia, United States; George Mason University, Fairfax, Virginia, United States; George Mason University, Fairfax, Virginia, United States; George Mason University, Fairfax, Virginia, United States

## Abstract

**OBJECTIVE:**

The Mason CARES study examined the effects of a randomly assigned personalized music intervention (Phase II) for care partners of older adults living with dementia after implementation of a 9-week virtual stress management program (Phase I) to reduce stress and increase well-being.

**METHODS:**

A total of 99 care partners completed the 20-week study across three cohorts in 2022. Baseline data on care partner stress levels and well-being were collected before beginning a 9-week stress management program (Phase I). Care partners were randomly assigned to a 4-week music intervention treatment or control group before the next stage of the intervention (Phase II). Researchers assisted care partners with identifying their family member’s favorite music during early adulthood years. MP3 players loaded with the favorite music were mailed to treatment group members. Focus groups were conducted to explore the utilization of personalized music. RESULTS: Nearly half (49%) were spousal care partners, ranging from 35-85 years old with 80% having at least a 4-year college degree, and 62% having an income greater than $70,000. Using ANOVAs to analyze mean stress levels and well-being, no significant differences in stress and well-being were found between the treatment and control groups. CONCLUSION: Despite the lack of statistically significant findings, preliminary analysis of focus group interviews revealed that many care partners benefited from the personalized music intervention. Effectiveness may depend on other factors, such as familiarity with using the technology, past experiences with music, care partner’s baseline level of stress, and the older adult’s stage of dementia.

